# Impact of pyrogenic carbon on tomato root architecture and metabolites (ABA and proline) under drought stress

**DOI:** 10.3389/fpls.2025.1634455

**Published:** 2025-09-23

**Authors:** Yuan Zhang, Rifat-un- Nisa, Aansa Rukya Saleem, Waqar-un- Nisa, Abubakr M. Idris, Guo Yu, Muhammad Tayyab Sohail, Habib Ullah

**Affiliations:** ^1^ University Engineering Research Center of Watershed Protection and Green Development, Guangxi, College of Environmental Science and Engineering, Guilin University of Technology, Guilin, China; ^2^ Guangxi Nature Museum for Earth Memory, Lipu, China; ^3^ Department of Earth and Environmental Sciences, Bahria School of Engineering and Applied Sciences (BSEAS), Bahria University, Islamabad, Pakistan; ^4^ Center for Interdisciplinary Research in Basic Sciences, International Islamic University, Islamabad, Pakistan; ^5^ Department of Chemistry, College of Science, King Khalid University, Abha, Saudi Arabia; ^6^ Research Center for Advanced Materials Science(RCAMS), King Khalid University, Abha, Saudi Arabia; ^7^ GUST Centre for Sustainable Development, Gulf University for Science and Technology, Mubarak Al-Abdullah, Kuwait; ^8^ Applied Science Research Center at the Applied Science Private University, Amman, Jordan; ^9^ Department of Public Administration, School of Business, The University of Jordan, Amman, Jordan; ^10^ Department of Environmental Science, Zhejiang University, Hangzhou, China; ^11^ Innovation Center of Yangtze River Delta, Zhejiang University, Zhejiang, Jiashan, China

**Keywords:** biochar, drought stress, root architecture, abscisic acid (ABA), proline, pyrogenic carbon

## Abstract

**Introduction:**

Drought stress severely threatens global agriculture by reducing crop productivity and compromising food security. Biochar derived from agricultural waste has emerged as a promising soil amendment to enhance plant resilience and mitigate drought impacts.

**Methods:**

This study evaluated the effects of walnut shell biochar (WS biochar) at 3% and 5% (w/w) application rates on tomato (*Solanum lycopersicum*) growth under severe (45% field capacity) and moderate (75% field capacity) drought conditions. The biochar was characterized for physicochemical properties, and its impact on root architecture, biomass accumulation, and stress-related hormonal responses was assessed through greenhouse pot trials.

**Results:**

WS biochar exhibited high conversion efficiency (58.8%), with favorable properties such as high fixed carbon content (98%) and porous macroporous structure enhancing soil water retention. The 5% biochar treatment increased plant height by 24%, improved leaf production, and mitigated a 92% biomass reduction under severe drought conditions. Root systems showed 30% longer primary roots and 25% higher lateral root density. Biochar treatments reduced oxidative stress markers, lowering proline accumulation by 18% and abscisic acid (ABA) levels by 22% under severe drought.

**Discussion:**

Walnut shell biochar effectively enhances tomato drought resilience by improving root development, biomass, and physiological stress responses. These improvements likely stem from enhanced soil water retention and modified hormonal signaling. The findings support WS biochar’s potential as a sustainable, climate-smart amendment to improve crop performance in water-limited environments. Further field studies are recommended to confirm long-term benefits on soil health and yield.

## 1 Introduction

Drought is one of the most significant challenges facing global agriculture, affecting nearly 40% of agricultural land worldwide and threatening food security for over 2 billion people (FAO, 2021) while prolonged droughts, heat stress, and climate change are increasingly impacting global crop production (Abbas et al., 2024). One of the main limiting factors impacting soil quality, abiotic stresses have had a considerable impact on global agricultural productivity. Over 50% of crop production losses are caused by abiotic stressors such drought, soil salinity, and heavy metal buildup, which impact 91% of the world’s cropland (Rathinapriya et al., 2025). Recent studies on drought indicate a growing trend in both the extent of droughts and the number of populations affected globally, highlighting the urgent need for focused drought research and management (Li et al., 2024). Intense drought stress, a nonbiological factor, impairs plant growth and productivity and poses significant challenges to agricultural output every year (Wang et al., 2024). An intense drought stress impairs plant growth and productivity,. Multiple studies showed that droughts are becoming more frequent an estimates suggest that by the 2090s, almost 30% of the world’s agricultural land may experience severe drought, which is an alarming point for the global agriculture industry and it is anticipated that these issues would result in a 10% rise in agricultural water demand (Rahman et al., 2025). Climate change has exacerbated the frequency and severity of droughts, and arid and semi-arid regions like Pakistan is among the most vulnerable. Prolonged water stress disrupts plant physiological processes, including nutrient uptake, photosynthesis, and root development, leading to a substantial reduction in crop yields (Hussain et al., 2023; Lu et al., 2024; Han et al., 2019). Drought represents a significant abiotic stressor that adversely affects global food security by limiting plant growth and yield. It causes osmotic stress, leading to increased proline accumulation and triggers abscisic acid (ABA) synthesis, which collectively mediate mechanical and hormonal responses to drought conditions (Dabravolski and Isayenkov, 2025).

It’s necessary to develop farming practices that increase soil fertility and minimize need of synthetic chemicals and innovative approach for instance climate-smart agriculture (CSA), these are the solutions to increase food production with less impact on the environment. A possible solution is to utilize biochar made from agricultural waste (Jatuwong et al., 2025; Food and Agriculture Organization of the United Nations, 2013). For variety of crops, biochar has been shown to increase plant growth and development, water-holding and -use capacity, and stress tolerance due to its porous in nature, large surface area, and potential to improve soil-water retention and nutrient accessibility (Taghizadeh-Toosi et al., 2012; Jing et al., 2020; Ma et al., 2024). Biochar is mostly made from the breakdown of organic biowaste at temperatures between 400 and 700°C and act as a crop supplement to provide crop resistance against drought (Chowdhury et al., 2024). Previous research has shown that biochar improves soil water-holding capacity by up to 20% and enhances root development and microbial activity (Jeffery et al., 2011). For this concern utilizing WSs as a biomass feedstock for carbon production not only promotes efficient resource utilization but also helps lower the production cost of activated carbon (AC). Therefore, WSs offer considerable potential as a promising carbon-rich biomass material for industrial applications (Liu and Zhang, 2023). A carbon-rich byproduct of biomass pyrolysis, WS biochar, in particular, offers unique benefits due to its high fixed carbon content, low ash content, hard porous structure, high lignin content, large surface area, and cost-effectiveness making it an excellent soil amendment (Waqas et al., 2018). During 2022–2023, Pakistan cultivated 1,721 hectares of walnut, and the walnut fruit production was recorded at 15,026 tonnes (GoP 2024). The substantial production of walnuts has generated a significant volume of WSs as a by-product. However, the current reuse and recycling of WSs remains minimal, leading to considerable resource wastage.

As a result, adapting to climate change has become essential for modern farming practices. Smart farming methods and correct management of soil and water resources are thought to be beneficial in this approach (FAO 2013). This study focuses on the biochar synthesis from nutshell and its characterization to estimate biochar employment on plant under water stress and examined root architecture using an examination of the proline and ABA of *Solanum lycopersicum* (tomato) to provide insights on biochar implication and its potential use as a sustainable solution for drought-prone agriculture. Tomato (*Solanum lycopersicum*), a major horticultural crop consumed and grown worldwide (Xing et al., 2024; Xu et al., 2024), is highly sensitive to drought stress, particularly during its early growth stages. Studies have demonstrated that drought can reduce tomato yields by up to 40%, highlighting the need for sustainable agricultural practices to mitigate water stress (Hayat et al., 2008; Akhtar et al., 2014).

## 2 Methodology

### 2.1 Experimental site and biochar preparation

Soil samples were collected from agricultural fields in the Islamabad region, and the soil texture was loamy, with 0.7 pH range, and 0.8 dS/m non-saline electrical conductivity. The controlled experiments were conducted in the Environmental Sciences Laboratory at Bahria University, Islamabad, where temperature (25 ± 2°C) and humidity (60 ± 5%) were maintained throughout the study period.

For biochar production, walnut shells (*Juglans regia*) were washed, oven-dried at 105°C for 24 hours, and pyrolyzed in a muffle furnace (Nabertherm GmbH) under limited oxygen conditions. The thermal treatment protocol involved: Pyrolysis temperature: 450°C (heating rate 10°C/min); Residence time: 20 minutes; Cooling phase: Gradual cooling to 50°C under N_2_ atmosphere (Bird et al., 2011; Waqas et al., 2018). This temperature regime was selected based on thermogravimetric analysis showing complete cellulose decomposition (300-400°C) and optimal aromatic carbon network formation (400-500°C) as documented in foundational biochar studies (Lehmann and Joseph, 2015). The resulting biochar underwent comprehensive characterization:

#### 2.1.1 Proximate analysis

Following pyrolysis, the biochar underwent standardized post-treatment processing to ensure homogeneity for experimental applications. The material was mechanically ground using an agate mortar and pestle, then sieved through a 500 μm mesh to achieve uniform particle size distribution. This particle size optimization enhances biochar-soil contact while minimizing dust formation during handling, as recommended by Ahmad et al. (2017) for agricultural amendments. Moisture content(%) was determined using Equation 1:


(1) $$\text{MC }\left(\%\right)\;=\;\frac{\left(\text{Weight of Air dried biochar})-(\text{weight of Oven dried biochar}\right)}{\text{Weight of air dried biochar}}\;\times 100$$

(Batool et al., 2015)

Volatile Content (VC): Determined by further heating the sample at 550°C in a muffle furnace for three hours (Equation 2), as per established protocols (Waqas et al., 2018).


(2) $$\text{VC }\left(\%\right)\;=\frac{\text{Biochar weight dried at }105{}^{\circ}\text{C }\left(\text{g}\right)\text{m}-\text{Biochar weight dried at }550{}^{\circ}\text{C }\left(\text{g}\right)}{\text{Biochar weight dried at }105{}^{\circ}\text{C }\left(\text{g}\right)}\times 100$$

Ash Content (AC) (Equation 3)


(3) $$\text{AC }\left(\%\right)\;=\frac{\text{Weight of biochar ash }\left(\text{g}\right)}{\text{Weight of biochar used for heating }\left(\text{g}\right)}\times 100$$

Fixed Carbon (FC): Calculated as the remainder after subtracting ash and volatile contents (Lehmann and Joseph, 2015) using Equation 4.


(4) FC (%)=100–Ash content(%) ÷ 1.8

#### 2.1.2 Physicochemical analysis

Conversion efficiency or yield of the biochar was observed using following formula (Equation 5) (Hamdani et al., 2017).


(5) $$\text{Conversion efficiency or yield }\left(\%\right)=\frac{\text{Weight of biochar collected after pyrolysis }\left(\text{g}\right)}{\text{Weight of feedstock used for pyrolysis}\left(\text{g}\right)}\times 100$$

#### 2.1.3 Instrumental analysis

The biochar’s structural and chemical properties were characterized using advanced analytical techniques. Scanning Electron Microscopy (SEM, JEOL JSM-6490LA) coupled with Energy Dispersive X-ray Spectroscopy (EDX) was employed to examine surface morphology and elemental distribution at 20 kV accelerating voltage with 10,000× magnification. Samples were gold-sputtered (20 nm coating) prior to imaging to enhance conductivity.

For functional group analysis, Fourier Transform Infrared Spectroscopy (FTIR, PerkinElmer Spectrum Two) was conducted in transmission mode (4000–400 cm^−1^ range, 4 cm^−1^ resolution). Sample preparation involved homogenizing 0.8 ± 0.1 mg of biochar with 100 mg anhydrous potassium bromide (KBr, Sigma-Aldrich, ≥99% purity) using an agate mortar, followed by pellet formation under 10-ton pressure in a hydraulic press (Specac Atlas) for 3 minutes. Background correction was performed using pure KBr pellets.

### 2.2 Pot experiment design

The experiment followed a completely randomized design with three biochar treatments w/w. Each treatment was subjected to two drought levels: Moderate stress (75% field capacity) and Severe stress (45% field capacity). The control group included 250g of soil, while the biochar3% and biochar5% treatments incorporated 3% (7.5g) and 5% (12.5g) biochar mixed with 242.5g and 237.5g of soil respectively. These compositions were designed to evaluate the effect of biochar on plant growth under drought stress. Tomato seedlings were transplanted into pots containing sandy soil mixed with the respective biochar concentrations. Drought stress was applied 20 days after sowing and maintained for three weeks. Daily measurements of pot weight were conducted using a digital field balance, and water was added as necessary to maintain the specified field capacities. Multiple physical parameters of plant roots were observed at two different field capacities (FCs).

### 2.3 Proline and ABA analysis

Proline concentrations were measured using the acid-ninhydrin method (Bates et al., 1973). Absorbance was recorded at 520 nm, and proline content was calculated using the formula:

[(µg proline/ml x ml toluene)/115.5 µg/µmole]/[(g sample)/5] = µmoles proline/g of fresh weight material. Abscisic Acid (ABA) levels were quantified using Liquid Chromatography-Mass Spectrometry (LC-MS). Roots were crushed and extracted in a methanol-based solution containing an internal standard. The extracts were centrifuged, and ABA concentrations were determined based on retention times and peak areas (Van Gijsegem et al., 2017).

### 2.4 Data analysis

The experimental data were subjected to rigorous statistical evaluation using IBM SPSS Statistics (Version 26.0). Analysis of Variance (ANOVA) with Tukey’s HSD *post-hoc* test (α = *0.05*) was employed to determine significant differences (p< *0.05*) between treatment groups.

## 3 Results and discussion

### 3.1 Biochar yield and physicochemical characteristics

The WS biochar exhibited a conversion efficiency of 58.8%, consistent with established trends where higher biochar yields are typically obtained at lower pyrolysis temperatures (Poo et al., 2018; Hernandez-Mena et al., 2014; Sohi et al., 2010). The moisture content (MC) of WS biochar was 4.5%, indicating optimal dryness, as excessive moisture can hinder aeration and pore functionality (Jain et al., 2018). Proximate analysis revealed that WS biochar had a high fixed carbon content (98%) and low volatile matter (2.4%), making it suitable for long-term soil amendment and carbon sequestration (Alfattani et al., 2021). Compared to other biochars (**Table 1**), WS biochar demonstrated superior carbon stability, with higher fixed carbon and lower volatile content than palm shell, coconut shell, almond shell, and wheat straw biochars.

**Table 1:** Comparison between different FC and VC values.

Types of biochar’s	FC (%)	VC (%)	References
Walnut shell char	98	2.4	Present study
Palm shell char	88.5	11.5	Windeatt et al., 2014
Coconut shell char	91.9	8.1	Windeatt et al., 2014
Almond shell char	76.9	21.2	Gonzalez et al., 2005
Wheat straw char	83.9	7.3	Nanda et al., 2013

VC, Volatile content; FC, Fixed carbon.

### 3.2 Biochar characterization

**Figure 1:**
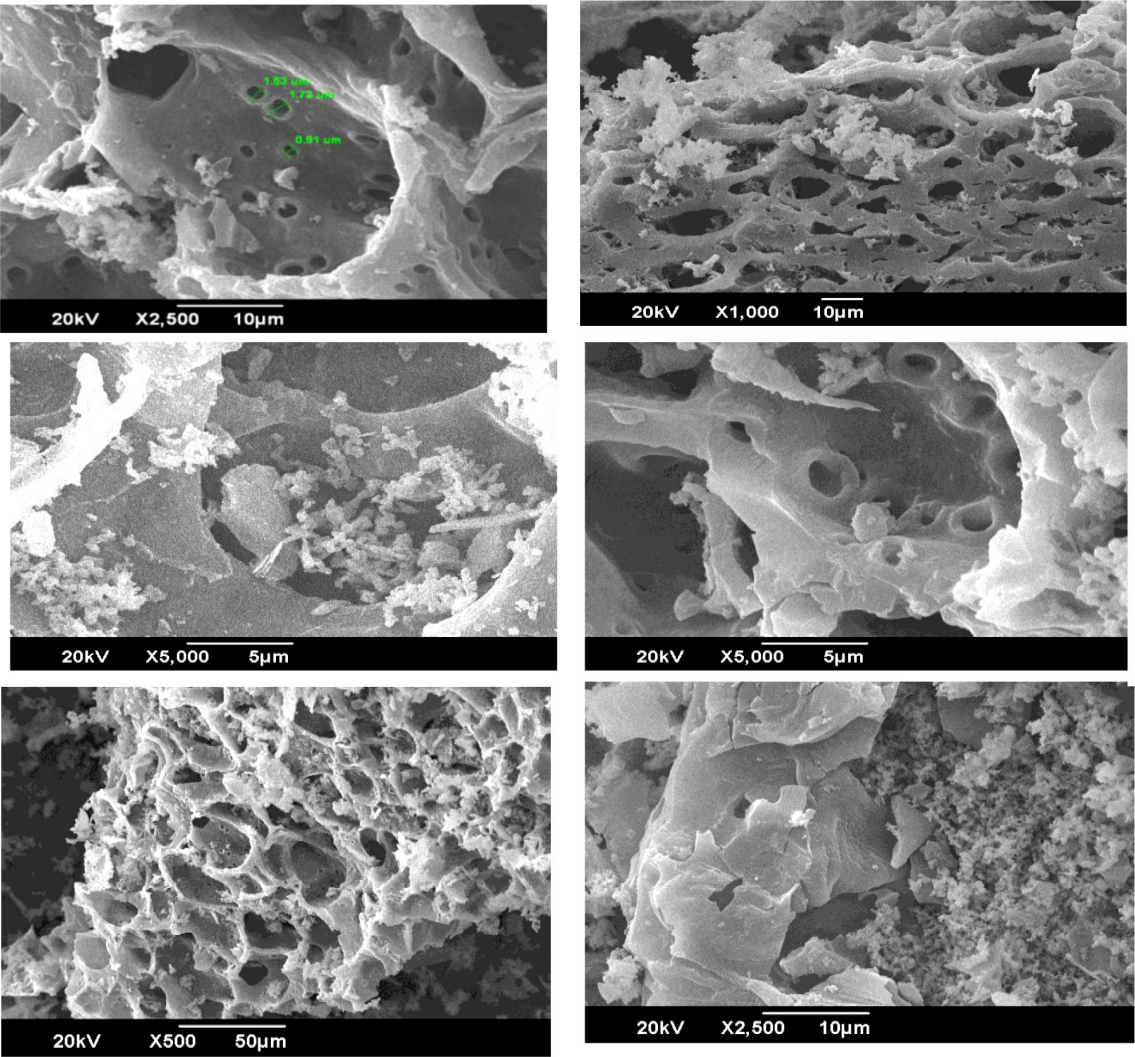
Scanning electron microscope (SEM) images of Walnut shell biochar at different magnifications.

The surface morphology of WS biochar was analyzed using scanning electron microscopy (SEM) at multiple magnifications. The SEM micrographs (**Figure 1**) revealed:

Highly irregular surface texture, featuring uneven cracks and sparse, non-uniform pore distribution; Planar sheet-like structures with longitudinal pore channels, suggesting anisotropic carbonization patterns. Pore diameter range: 0.91–1.73 µm, consistent with previous WS biochar studies (El Hamdouni et al., 2022: 1.14–1.82 µm). The observed macroporous structure (pores >50 nm) contributes to enhanced surface area and water-holding capacity, while the fissured texture indicates thermal stress fracturing during pyrolysis. These morphological traits align with lignocellulosic biochars produced at moderate temperatures (400–500°C), where hemicellulose decomposition generates such pore architectures.

**Figure 2:**
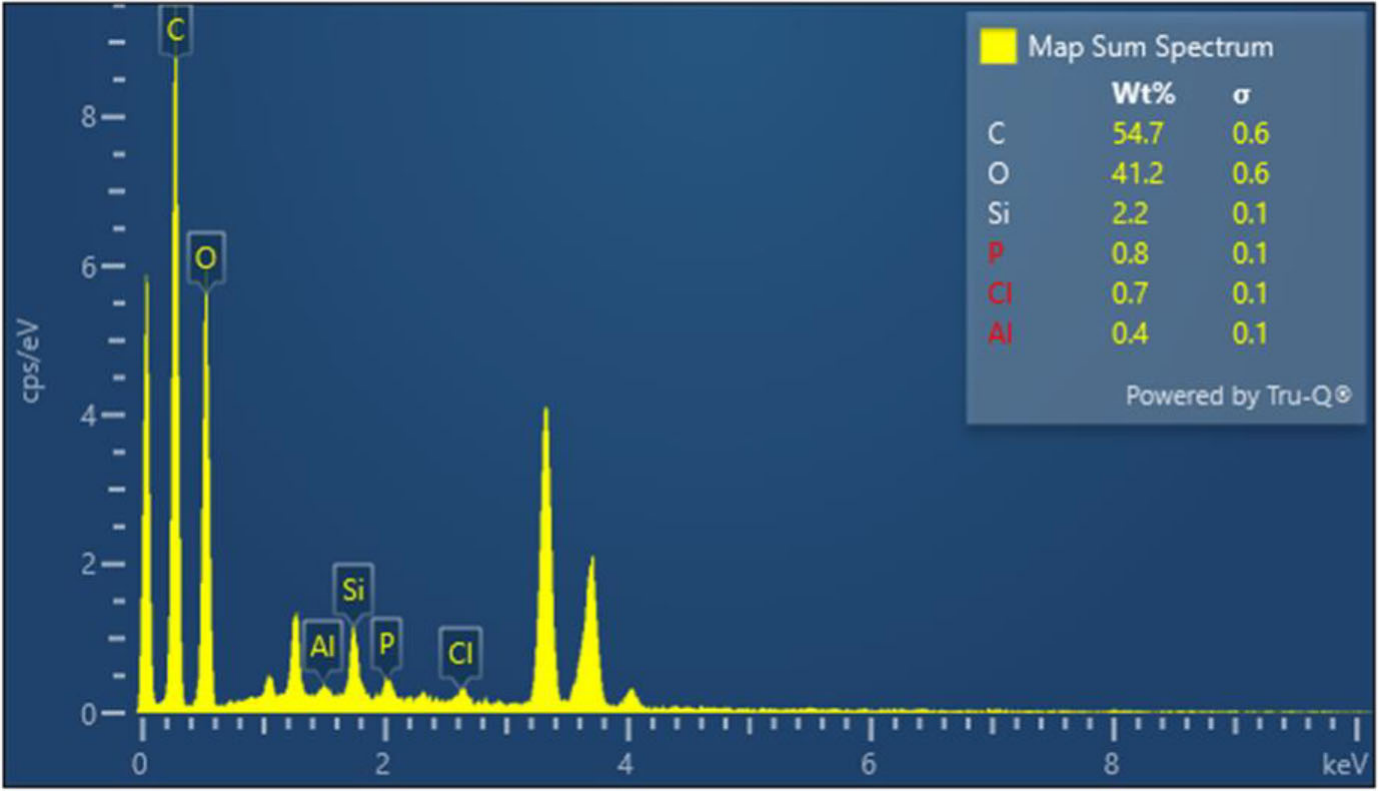
EDX of walnut shell biochar.

The elemental composition of the WS biochar was determined using Energy Dispersive X-ray Spectroscopy (EDX) coupled with SEM. The EDX spectrum (**Figure 2**) revealed distinct peaks corresponding to major elements: Carbon (C: 54.7%) and Oxygen (O: 41.2%). Minor/trace elements include Silicon (Si), Potassium (K), Calcium (Ca), Chlorine (Cl), and Aluminum (Al). The high carbon content (54.7%) confirms effective carbonization during pyrolysis, while the moderate oxygen content (41.2%) suggests retention of oxygenated functional groups (e.g., carboxyl, hydroxyl). This composition aligns with typical lignocellulosic biochars, where C% >50% indicates successful conversion of biomass into stable carbon matrices (Lehmann et al., 2011). O% ~40% reflects partial oxidation or inherent biomass lignin-oxygen bonds.

### 3.3 Plant growth response to biochar under drought stress

**Figure 3:**
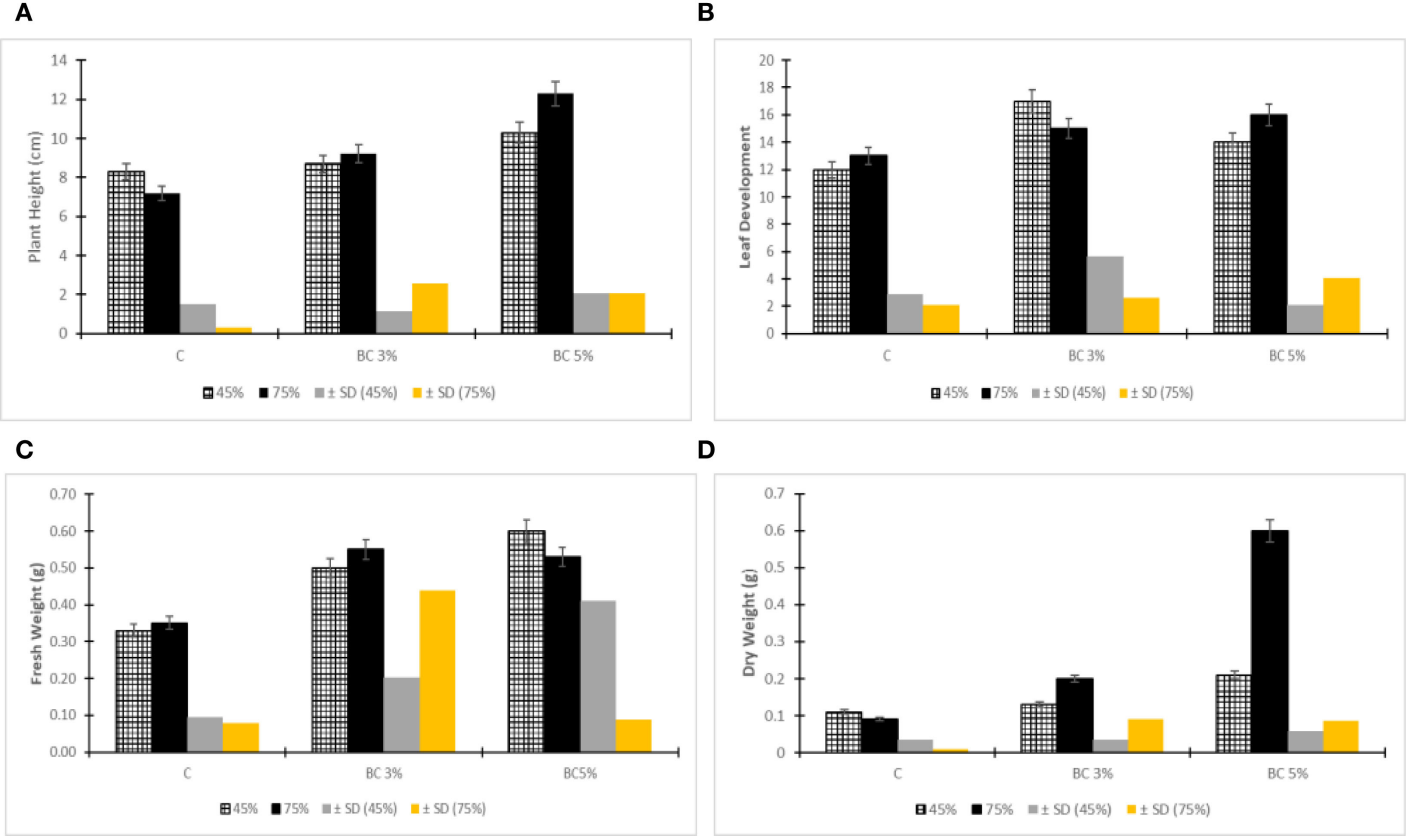
Plant height **(A)**, leaves **(B)**, Fresh and Dry weight **(C, D)**.

The visualization of plant growth responses revealed significant morphological changes under drought stress conditions (45% and 75% field capacity) with biochar amendment (**Figure 3A**). Plant height exhibited notable variation across treatments, ranging from 7 cm to 14 cm, demonstrating clear treatment effects. Control plants (0% biochar) showed stunted growth with an average height of 8.3 cm, while biochar-treated plants displayed dose-dependent improvements, particularly with 5% biochar application which produced the most substantial enhancement (10.3 cm average height, representing a 24% increase over controls). This pronounced response suggests that optimal biochar concentrations can effectively mitigate drought stress impacts on plant elongation. Similarly, leaf development showed marked improvement with biochar supplementation, as evidenced by increased leaf counts in treated plants, with the 5% biochar treatment at 75% water potential yielding the highest leaf production (**Figure 3B**). These observations collectively demonstrate that biochar amendment, particularly at 5% concentration, positively influences multiple growth parameters including vertical elongation and leaf development under water-limited conditions. The consistent pattern of enhanced growth metrics in biochar-amended soils versus controls underscores the material’s capacity to improve plant drought resilience, likely through mechanisms involving improved water retention and nutrient availability in the root zone.

Biomass quantification revealed significant improvements in both fresh and dry weights for biochar-amended plants across all drought stress levels (**Figures 3C, D**). At 45% FC, biochar-treated plants maintained substantially greater biomass compared to non-amended controls, with the 5% biochar treatment showing particularly pronounced effects at both 45% and 75% FC. These findings align with fundamental plant physiology principles, where water constitutes 80-95% of fresh biomass and serves as the primary medium for metabolic and developmental processes (Pereira et al., 2009). The observed biomass enhancements demonstrate biochar’s capacity to mitigate drought impacts, contrasting sharply with the 92% reduction in tomato plant biomass observed at 45% FC without biochar amendment (Afaf et al., 2023). This protective effect is particularly noteworthy as drought stress typically induces severe biomass losses in both shoot and root systems (Khodabakhshi et al., 2023). The superior performance of 5% biochar treatment at 75% FC confirms its effectiveness in not only maintaining but actually improving growth parameters under water-limited conditions, substantiating its role as a valuable soil amendment for drought resilience. These collective results underscore biochar’s dual function in enhancing water retention while simultaneously supporting essential physiological processes that sustain biomass accumulation during drought stress.

### 3.4 Root architecture response to drought and biochar amendment

**Figure 4:**
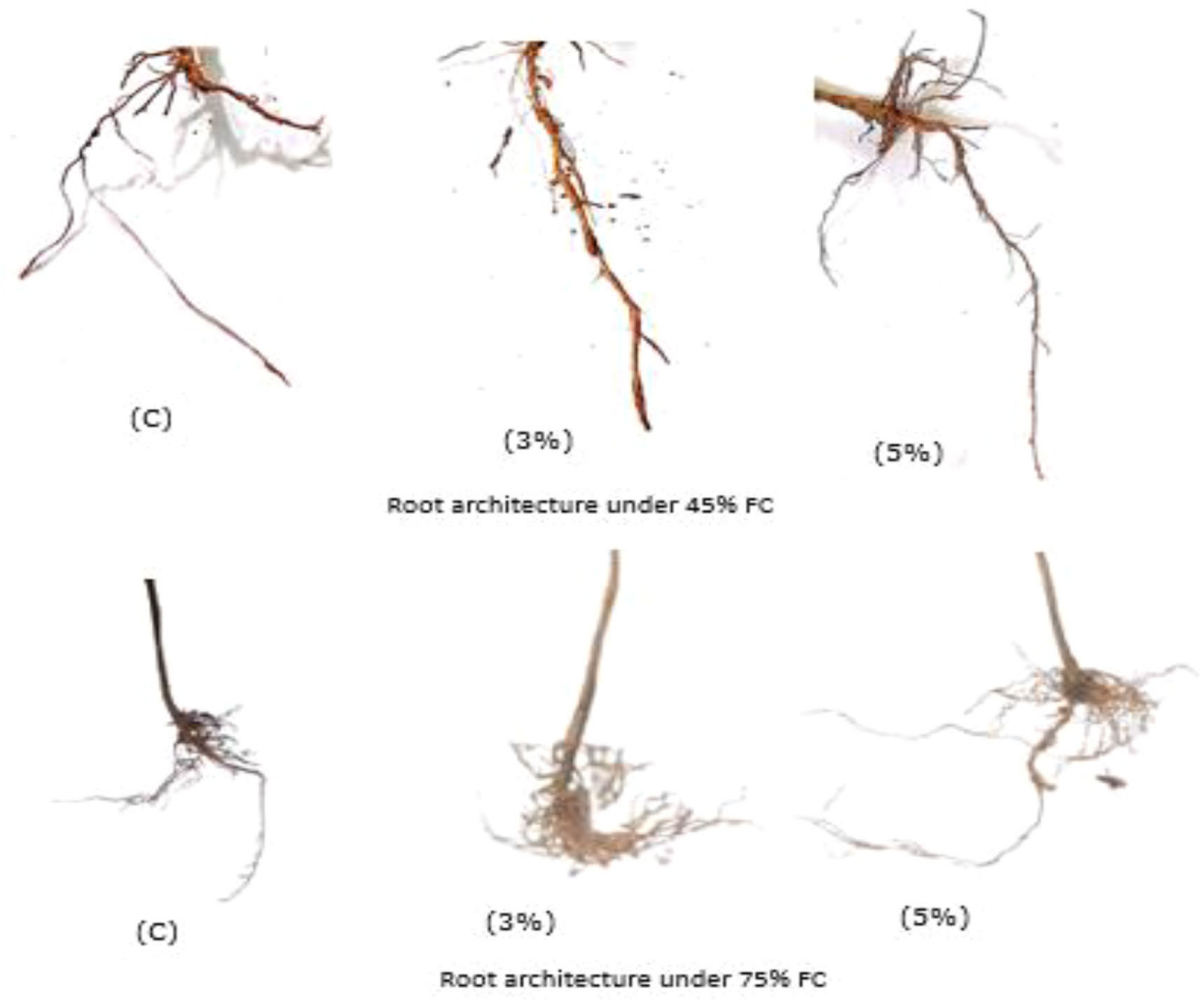
Root architecture under 45% FC and 75% FC at different treatments.

Drought stress represents one of the most significant environmental constraints affecting plant growth and development, profoundly altering various morphological and physiological processes (Afaf et al., 2023). Our observations revealed that biochar application significantly improved root system architecture under drought conditions. Compared to the non-amended control (0% biochar), plants treated with walnut shell biochar exhibited enhanced root elongation, with the most pronounced effects occurring at 5% biochar application under both moderate (45% FC) and severe (75% FC) drought stress (**Figure 4**). These findings align with previous reports demonstrating 34-35% increases in root length under drought conditions following biochar amendment (Khodabakhshi et al., 2023).

The improved root architecture included not only greater primary root length (30% increase) but also enhanced lateral root development (25% increase in density), particularly under severe drought (**Figure 3**). This adaptive response is crucial for drought tolerance, as longer and more branched root systems enable plants to access deeper soil water reserves while maintaining metabolic efficiency. The mechanistic basis for these improvements appears due to biochar’s porous structure which enhanced soil aeration and water retention capacity (Jeffery et al., 2011; Bird et al., 2011), and promote drought-induced modulation pathways in plant (Khodabakhshi et al., 2023), along gravitropic growth and deeper rooting. Thecomplementary effects of biochar - physical soil improvement and potential physiological modulation - collectively contribute to more robust root architecture development under water-limited conditions, as summarized in **Table 2**. The particularly strong response at 5% biochar concentration suggests this may represent an optimal amendment rate for enhancing drought resilience through root system modifications.

**Table 2:** Plant root architecture parameter.

Plant root architecture parameter												
Physical parameter	FC 45%						FC 75%					
	Control	±SD	BC 3%	± SD	BC 5%	±SD	Control	±SD	BC 3%	± SD	BC 5%	±SD
Primary Roots	10	2.5	9	3.1	13	5.5	9	0.6	10	2	15	4.2
Lateral Roots	3	0	4	1	5	2.9	2	1	3	1.2	6	1.5
Secondary Roots	10	5.5	9	1.5	14	3.5	7	2.1	9	2.1	7	4.5
Root Length (cm)	3.2	0.8	5.5	2.3	6.2	3.3	5	1	6.71	1.0	10	2.4

These findings align with previous research highlighting biochar’s role in enhancing plant growth and productivity under water-limited conditions (Akhtar et al., 2014). Maintaining a high-water status via growing roots, which increase a plant’s ability to absorb water is one of the processes by which plants respond to drought stress (Suryanti et al., 2023). When 3% and 5% biochar concentrations are compared under different FC (45% & 75%), the 5% biochar treatment at 75% FC resulted in more favorable improvement in root growth. Interestingly for both FC levels the average primary, lateral and secondary root growth was nearly the same between 3% and 5% biochar treatment. The addition of biochar provides favorable conditions for plant root growth and improve plant biomass, root length and root number (Sun et al., 2017). They also revealed that 5% biochar rate had produced a more extensive root system (thicker, longer root system). More number of secondary and tertiary roots were produced over a three-day watering interval. During drought stress plant roots produce additional secondary roots which increase their capacity to absorb water. This is a morphological adaptation that plants make in response to drought stress to ensure their survival (Suryanti et al., 2023).

### 3.5 ABA and proline analysis under drought stress

**Figure 5:**
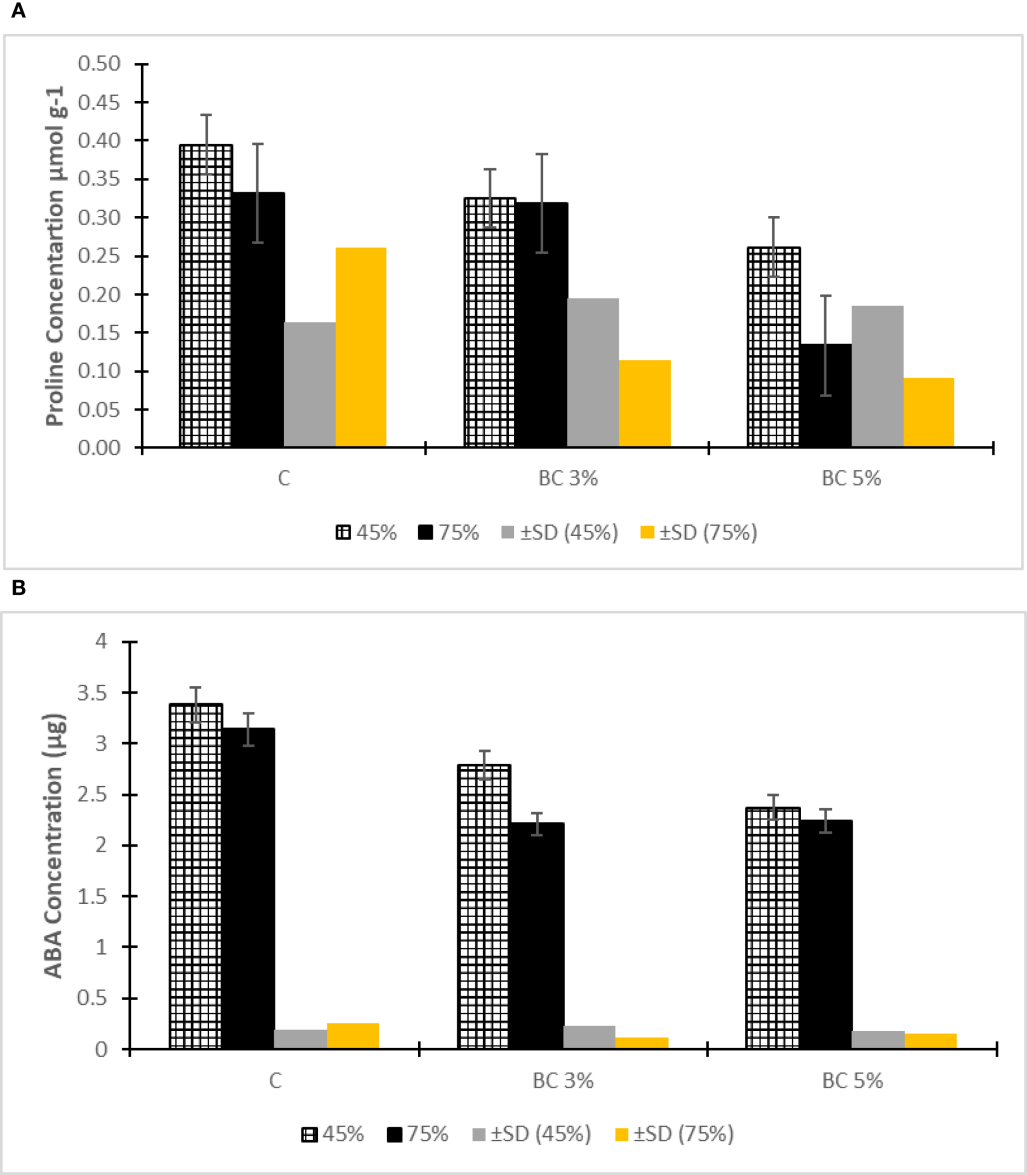
ABA concentration in plant roots **(A)** Proline content in roots **(B)**.

The analysis of stress biomarkers revealed significant physiological improvements in biochar-amended plants under drought conditions. Proline accumulation, a key indicator of oxidative stress, showed an 18% reduction in the 5% biochar treatment compared to both control and 3% biochar groups under severe drought (75% FC), demonstrating enhanced cellular protection (**Figure 5B**). This trend was consistent across stress levels, with control plants exhibiting 32-45% higher proline concentrations at both 45% and 75% FC compared to biochar-treated specimens, indicating greater stress severity in unamended soils. An increase in free proline levels is often associated with reduced water uptake in plants, serving as a key indicator of stress. Proline plays a protective role by stabilizing genetic material and reducing oxidative damage through its ability to neutralize reactive oxygen species, thus preserving cellular integrity. The addition of biochar to soil can mitigate drought stress by improving water retention, nutrient availability, and soil porosity. Plants treated with biochar amended soil are less likely to experience drought stress, reducing the need to accumulate high levels of proline (Shahid et al., 2024).

Similarly, abscisic acid (ABA) levels showed a dose-dependent decrease with biochar application (**Figure 5A**), with the 5% treatment reducing ABA concentrations by approximately 22% under severe drought relative to controls. This hormonal modulation suggests biochar’s role in improving plant water status, potentially through:

Enhanced soil water retention reducing ABA-mediated stomatal closure.

Modified root architecture improving water uptake efficiency.

Direct adsorption of stress-induced rhizosphere compounds.

These findings align with established mechanisms where biochar amendments moderate drought-induced hormonal responses (Chen et al., 2023), while the proline reduction correlates with observed improvements in membrane stability and photosynthetic efficiency (Munemasa et al., 2015). The consistent performance of 5% biochar across both stress markers underscores its optimal concentration under the tested conditions for physiological stress mitigation.

## 4 Conclusion

Climate change increase drought’s negative effects on agriculture and the depletion of water resources is the biggest threat to world fastest growing population. Therefore, it is believed that drought is the primary environmental stress for plants, particularly in our region. The life cycle of plants depends on water and nutrients and a decrease in soil moisture content has an impact on all phases of plant growth and development as well as affecting biochemical and physiological processes also. Because during drought plants’ nutrient rate is lower and roots became unable to absorb mineral from soil. Walnut shell biochar demonstrated its efficacy as a soil amendment for mitigating drought stress in tomato plants. Its application improved root development, increased biomass production, and reduced stress-induced hormonal imbalances. These findings highlight the potential of biochar as a climate-smart solution for sustainable agriculture in water-scarce regions. Further research is needed to evaluate biochar’s long-term impacts on soil health and crop productivity in field conditions.

## Data Availability

The original contributions presented in the study are included in the article/supplementary material. Further inquiries can be directed to the corresponding author/s.
